# Epidemiological role of novel and already known ‘*Ca*. P. solani’ cixiid vectors in rubbery taproot disease of sugar beet in Serbia

**DOI:** 10.1038/s41598-023-28562-8

**Published:** 2023-01-25

**Authors:** Andrea Kosovac, Živko Ćurčić, Jelena Stepanović, Emil Rekanović, Bojan Duduk

**Affiliations:** 1 Laboratory of Phytopathology, Institute of Pesticides and Environmental Protection, 11080 Belgrade, Serbia; 2 Sunflower Department, Institute of Field and Vegetable Crops, 21000 Novi Sad, Serbia

**Keywords:** Pathogens, Plant sciences, Entomology

## Abstract

Rubbery taproot disease (RTD) of sugar beet was recently associated with the plant pathogenic bacterium ‘*Candidatus* Phytoplasma solani’ (CaPsol) and reported throughout the Pannonian Plain with variations in severity. Tracing CaPsol epidemiological pathways was performed in the experimental sugar beet field in Rimski Šančevi (Serbia) in 2020–2021, where an RTD outbreak was recently recorded. A molecular epidemiology approach was applied to the study of three RTD occurrence scenarios: epidemic, non-epidemic and ‘absence of RTD’. As a result, *Hyalesthes obsoletus ex Convolvulus arvensis* was detected as a CaPsol vector to sugar beet, while two other cixiids were identified for the first time as vectors of the CaPsol-induced plant disease in crops: *Reptalus quinquecostatus* and* R. cuspidatus*. *R*. *quinquecostatus* was proposed culpable for the 2020 RTD epidemic outbreak in Rimski Šančevi when dSTOLg CaPsol strain predominated in the RTD-affected sugar beet, whereas *R. cuspidatus* had a negligible role in RTD occurrence and displayed ambiguous involvement in CaPsol epidemiology on a wider scale. The temporal discrepancy of the offset of CaPsol dissemination and disease occurrence is the main obstacle in predicting CaPsol-induced diseases. Predicting disease occurrence and severity can only be achieved by gaining a better understanding of CaPsol epidemiological pathways and insect vectors involved in disease outbreaks.

## Introduction

‘*Candidatus* Phytoplasma solani’ (CaPsol) is a phytopathogenic bacterium that affects numerous cultivated plants throughout Europe^1,2^. Rubbery Taproot Disease (RTD) of sugar beet is the most recently described disease caused by CaPsol, the etiology of which was revealed during a disease outbreak in 2018/2019 in Rimski Šančevi (Serbia)^3^. The sugar beet cultivation area in Serbia affected with RTD is situated in the country’s north where the vast majority of this crop is grown (97%), covering − 50.000 ha with an average production of 2.5 MT per year (FAOSTAT) ^4^. Typical RTD symptoms are rubbery taproots, as well as yellowing, wilting and necrosis of leaves^3,4^. RTD causes significant yield losses and difficulties in further industrial processing of the rubbery taproots. Furthermore, affected taproots are prone to rotting by secondary and opportunistic fungal pathogens either in fields or in clamps. A survey for RTD throughout the Pannonian Plain, conducted in 2020, showed variations in disease severity from an almost unnoticed occurrence in Austria, to epidemics in Serbia and Slovakia^4^. These findings showed that the area of sugar beet production in Europe susceptible to CaPsol is significantly larger than its previously reported confinement to France^4,5^.

The epidemiology of CaPsol-induced plant diseases is largely dependent on the feeding preferences of insect vectors that feed on phloem sap, which are primarily planthoppers of the family Cixiidae (Hemiptera, Auchenorrhyncha)^6^. The most efficient CaPsol vector is the widely distributed cixiid planthopper *Hyalesthes obsoletus* Signoret, associated with CaPsol-outbreaks in several solanaceous crops and grapevine^7–12^. *Reptalus panzeri* (Löw) is another CaPsol vector that was initially associated with maize redness/corn reddening disease in Serbia, but its vector role was later extended to other crops^10,13,14^. Its congeneric species, *R. quinquecostatus* (Dufour) sensu Holzinger et al*.*^15^ was experimentally confirmed as a CaPsol transmitter to periwinkle, but not to crop plants^16^. A cixiid of particular interest in the context of sugar beet RTD is *Pentastiridius leporinus* (Linné), the only known vector of both, CaPsol and ‘*Candidatus* Arsenophonus phytopathogenicus’ to sugar beet, the latter being associated with the disease syndrome “basses richesses” (SBR) in France and Germany^5,17–19^.

Vectors disperse CaPsol from natural reservoirs—weeds and wild plants—some of which can be used by insects as their hosts^6,20^. The traditional epidemiological divergence of CaPsol strains, based on the house-keeping *tuf* gene (encoding factor tu), was originally attributed to host plant preferences of the vector *H. obsoletus*^21^. *Urtica dioica* L. hosts *H. obsoletus* populations and outsources CaPsol strains of tuf-a and tuf-b2 (tuf-ab) genotypes belonging to the tuf-a epidemiology^21–23^. Populations of *H. obsoletus* associated with *Convolvulus arvensis* L., *Crepis foetida* L. and *Vitex agnus-castus* L., disperse CaPsol strains of the tuf-b type in central and southern Europe^12,21,23,24^. Tuf-b1 genotype was further established as corresponding to the reference CaPsol STOL strain of the tuf-b type^1,22^, while three novel types/genotypes, tuf-b3, b5 and b6, were additionally described^25,26^. Molecular typing of CaPsol strains involved in RTD of sugar beet revealed another novel *tuf* sequence variant, tuf-d, bearing one SNP difference compared to tuf-b1^3^.

Previously found in nearly all RTD-affected sugar beet fields assessed throughout the Pannonian Plain, the CaPsol multilocus genotype dSTOLg (tuf-d/STOL/V2-TA), predominated in localities with epidemic RTD severity^4^. Therefore, the aim of this study was to reveal whether crop-specific CaPsol epidemic cycle(s) are involved in the RTD of sugar beet in Serbia, as well as whether these novel or previously known transmission routes can be associated with different RTD severities. Tracing CaPsol pathways through different hosts was achieved by a molecular epidemiology approach based on three genes: *tuf*, *stamp* (encoding an antigenic membrane protein) and *vmp1* (encoding a variable membrane protein), each expressing affiliation to either tuf-a or tuf-b epidemiological cycles^22–24,27,28^.

The RTD epidemiological study was conducted in 2020 and 2021 on the experimental sugar beet field of the Institute of Field and Vegetable Crops in Rimski Šančevi, where CaPsol was originally associated with RTD, and an epidemic disease occurrence was recorded in 2019^3,4^. Three different RTD severity occurrence scenarios were assessed on two sugar beet plots: (1) epidemic; (2) non-epidemic and (3) ‘absence of RTD’. All key points of the CaPsol epidemiology were examined: potential vectors, tentative reservoir plants and sugar beets in the field. Furthermore, CaPsol transmission trials were carried out via three putative cixiid vectors found in situ: *R. quinquecostatus *sensu Holzinger et al*.*^15^, *H. obsoletus ex C. arvensis* (*Ca*) and *Reptalus cuspidatus* (Fieber). In addition, the presence of **‘***Ca.* A. phytopathogenicus**’**, the causal agent of SBR, was assessed in all field-collected sugar beets.

## Results

### RTD severity evaluation and assessment of ‘*Ca.* P. solani’ and ‘*Ca.* A. phytopathogenicus’

Two sugar beet plots in the experimental sugar beet field in Rimski Šančevi, referred to as plot-1 and plot-2, were evaluated for the occurrence and severity of RTD in two years, 2020 and 2021. Epidemic RTD severity was evaluated in plot-1 in 2020 with a disease incidence of 10.3%^4^. Although symptomatic plants were scattered throughout the plot, a prominent aggregation of the sugar beet that was heavily affected by RTD was observed in the northeast (NE) plot section where the adjacent boundary strip hosted a dense weed area earlier in the season (Fig. S1). In 2021, RTD severity was evaluated in plot-1 as non-epidemic with an incidence of 0.1%, with RTD-symptomatic sugar beets only found scattered in the plot. No RTD symptoms were observed in sugar beet plot-2 in either 2020 or 2021. Typical SBR symptoms (e.g. discoloration of root vascular tissue) were not observed on either of the two sugar beet plots.

A total of 50 RTD-symptomatic sugar beets sampled from plot-1 (25 per year) were all CaPsol-infected. Thirty asymptomatic sugar beets collected from plot-2 (15 per year) were all CaPsol negative, confirming this plot as RTD-free in both years. All 80 field-collected sugar beets, RTD-symptomatic and asymptomatic, were negative in the ‘*Ca.* A. phytopathogenicus’ TaqMan qPCR assay, while the ‘*Ca.* A. phytopathogenicus’ positive control HN1220/5 showed strong amplification with C_q_ values < 32. All analyzed samples, as well as the positive control, also showed high amplification with C_q_ values < 25 in the plant (sugar beet) qPCR assay, confirming high content and quality of extracted DNA.

### Identification of tentative ‘*Ca.* P. solani’ reservoir plants

A total of 10 weed species were present in plot-1 in 2020 (Table 1). Only *C. arvensis* permeated the entire sugar beet plot and surrounding boundary strips, while the other weeds were aggregated on the NE boundary strip. In this area, weeds have grown through the crop edge, and spatially overlapped with the aggregation of RTD-symptomatic sugar beets reported above. Upon sampling, all weeds were asymptomatic. Up to 15 samples of each weed species were collected, with the exception of *C. arvensis* (25). The highest CaPsol infection rate of 60% was detected in *C. arvensis* and *Ambrosia artemisiifolia* L., followed by *Datura stramonium* L. (44%) and *Amaranthus retroflexus* L. (33%). *Solanum nigrum* L. and *Chenopodium album* L. each had 20% positive samples, while only 1/9 *Sorghum halepense* (L.) Pers. was infected with CaPsol. None of the *Chenopodiastrum hybridum* (L.) S. Fuentes, Uotila, & Borsch (*Chenopodium hybridum* L.), *Portulaca oleracea* L. and *Hibiscus trionum* L. samples were positive for CaPsol (Table 1).

**Table 1:** CaPsol multilocus genotypes detected in weeds in sugar beet plot-1 during the 2020 epidemic RTD occurrence.

Weed species	CaPsol positive/total no. of samples	CaPsol multilocus genotype *tuf*/*stamp*/*vmp1*	No. of samples	CaPsol strain	GenBank acc. no
*C. arvensis*	15/25	tuf-d/STOL(St4)/V2-TA	3	Ca635/20	OP231777^t^
		tuf-b1/Rqg31(St2)/V2-TA	5	Ca627/20	OP231775^t^
		tuf-b1/Rqg31/V4	3		
		tuf-b1/Rqg31/V14	1		
		tuf-b1/M5(St28)/V4	1	Ca634/20	OP231776^t^
		tuf-b1/M5/V14	2		
*A. retroflexus*	5/15	tuf-d/STOL(St4)/V2-TA	1	Ar1051/20	OP231784^t^
		n.a./STOL/n.a	1		
		n.a./RTD6(St76)/V23	1		
		n.a./**St79**/n.a	1	Ar1055/20	OP156883^s^
		n.a./**St80**/n.a	1	Ar1059/20	OP156884^s^
*Ch. album*	3/15	tuf-d/STOL(St4)/V2-TA	3		
*Ch. hibridum*	0/7	/	/		
*P. oleracea*	0/15	/	/		
*H. trionum*	0/10	/	/		
*D. stramonium*	4/9	n.a./STOL(St4)/n.a	1		
		n.a./Rqg50(St1)/n.a	1		
		tuf-b2/19–25(St11)/V18	1	Ds964/20	OP231778^t^
		n.a./**St78**/n.a	1	Ds958/20	OP156882^s^
*S. nigrum*	3/15	tuf-d/STOL(St4)/V2-TA	2	Sn1019/20	OP231780^t^
		tuf-b1/Rqg31(St2)/V4	1	Sn1018/20	OP231779^t^
*A. artemisiifolia*	9/15	tuf-d/STOL(St4)/V2-TA	7	Aa1079/20	OP231783^t^
		tuf-b1/Rqg31(St2)/V2-TA	1	Aa1085/20	OP231781^t^
		tuf-b1/M5(St28)/V14	1	Aa1089/20	OP231782^t^
*S. halepense*	1/9	n.a./STOL(St4)/n.a	1		

The “St” *stamp* sequence variants, corresponding to the original *stamp* genotype designation^28,29^, are provided in parentheses next to the first mention of a particular genotype in a given host plant. Newly detected *stamp* genotypes are labelled in bold. Affiliated “St” *stamp* sequence variants are elaborated in Supplementary Table S1; not amplified (n.a.); *stamp* sequence (^s^); *tuf* sequence (^t^).

### Cixiid community in an experimental sugar beet field

A cixiid survey conducted in 2020 and 2021 in sugar beet plot-1 revealed the sympatric presence of three cixiid species: *H. obsoletus* (*Ca*), *R. quinquecostatus* and *R. panzeri*. Another cixiid, *R. cuspidatus*, was found in 2021 in plot-2. The first individuals of *R. quinquecostatus* were detected on June 10th 2020 in the weed area on the NE boundary strip of plot-1. The population achieved its peak around June 20th and was very abundant during the following two weeks (30 individuals per 10 sweeps). Individuals of *R. quinquecostatus* were also found among the sugar beets in the NE section of plot-1, coinciding with the later revealed RTD hotspot. Individuals of *H. obsoletus* (*Ca*) appeared in plot-1 at the end of June and were present until July 20th, spatially coinciding with *C. arvensis* along the boundary strips and within plot. Therefore, the *H. obsoletus* population was dispersed and its abundance appeared lower than that of the *R. quinquecostatus* (5 individuals per 10 sweeps).

In 2021, *R. quinquecostatus* appeared in mid-June on the same NE boundary strip of plot-1, while in the expected period of highest abundance (June 20th), the number of collected individuals was significantly reduced compared to 2020 (0–1 individuals per 10 sweeps). In addition, *R. panzeri* was present syntopically with *R. quinquecostatus*. A total of 37 *Reptalus* spp. specimens were caught and identified by combining evaluations of morphology and a molecular marker, the ITS2 gene. *R. quinquecostatus* had majority presence of 68%, while the remaining individuals were identified as *R. panzeri* (Table 2). The population of *H. obsoletus* appeared in plot-1 in the same period as in the previous year and was again dispersed along the boundary strips and within the plot.

**Table 2:** CaPsol multilocus genotypes detected in putative cixiid vectors.

Cixiid species (plot/year)	CaPsol positive/total no. of samples	CaPsol multilocus genotype *tuf*/*stamp*/*vmp1*	No. of samples	CaPsol strain	GenBank acc.no
*R. quinquecostatus* (plot-1/2020)	38/57	tuf-d/STOL(St4)/V2-TA	16	Rq87/21	OP231765^t^
		n.a./STOL/n.a	9		
		n.a./Rqg31(St2)/V4	3		
		n.a./Rqg31/n.a	3		
		tuf-b1/BG4560(St31)/V7-A	3	Rq72/21	OP231764^t^
		n.a./RTD6(St76)/n.a	1		
		n.a./**St82**/n.a	1	Rq104/21	OP156886^s^
		n.a./**St83**/n.a	1	Rq403/21	OP156887^s^
		n.a./**St84**/n.a	1	Rq423/21	OP156888^s^
*H. obsoletus ex C. arvensis* (plot-1/2020)	26/62	tuf-d/STOL(St4)/V2-TA	4	HoCa233/21	OP231766^t^
		tuf-b1/STOL/V2-TA	2	HoCa430/21	OP231767^t^
		n.a./STOL/n.a	6		
		tuf-b1/Rqg31(St2)/V2-TA	4		
		n.a./Rqg31/n.a	1		
		tuf-b1/Rqg50(St1)/V4	1	HoCa230/21	OP231769^t^
		tuf-b1/Rqg50/V14	2		
		n.a./Rqg50/n.a	1		
		tuf-b1/M5(St28)/V2-TA	1	HoCa444/21	OP231770^t^
		tuf-b1/M5/V14	3		
		n.a./**St89**/n.a	1	HoCa238/21	OP156893^s^
*R. quinquecostatus* (plot-1/2021)	9/25	n.a./STOL(St4)/n.a	4		
		n.a./Rqg31(St2)/V2-TA	1		
		n.a./Rqg31/V4	1		
		n.a./**St85**/n.a	1	Rq1497/21	OP156889^s^
		n.a./**St86**/n.a	1	Rq1498/21	OP156890^s^
		n.a./**St87**/n.a	1	Rq1511/21	OP156891^s^
*R. panzeri* (plot-1/2021)	5/12	tuf-d/STOL(St4)/V2-TA	2	Rp1519/21	OP231774^t^
		n.a./STOL/n.a	1		
		tuf-b1/Rqg31(St2)/V2-TA	1	Rp1507/21	OP231773^t^
		n.a./**St88**/n.a	1	Rp1531/21	OP156892^s^
*H. obsoletus ex C. arvensis* (plot-1/2021)	20/30	tuf-b1/STOL(St4)/n.a	1		
		tuf-b1/Rqg31(St2)/V2-TA	3		
		tuf-b1/Rqg31/V4	1	HoCa92/22	OP231768^t^
		tuf-b1/Rqg31/V14	3		
		n.a./Rqg31/n.a	4		
		tuf-b1/Rqg50(St1)/V14	2		
		tuf-b1/Rpm35(St3)/V2-TA	1	HoCa94/22	OP231771^t^
		n.a./Rpm35/n.a	1		
		tuf-b1/M5(St28)/V14	2		
		tuf-b1**/St94**/V2-TA	1	HoCa90/22	OP156898^s^ OP231772^t^
		n.a./**St95**/n.a	1	HoCa99/22	OP156899^s^
*R. cuspidatus* (plot-2/2021)	8/35	n.a./STOL(St4)/n.a	5		
		n.a./M5(St28)/n.a	1		
		n.a./**St90**/n.a	1	Rc1451/21	OP156894^s^
		n.a./**St91**/n.a	1	Rc1470/21	OP156895^s^

Affiliated “St” *stamp* sequence variants are elaborated in Supplementary Table S1; not amplified (n.a.); *stamp* sequence (^s^); *tuf* sequence (^t^).

Evaluated in 2020 as an RTD-free plot, plot-2 was inspected for cixiids in 2021. The only cixiid found in this plot was *R. cuspidatus*, present on the ruderal vegetation in a ditch between the plot and the local road. The first *R. cuspidatus* individuals were collected in mid-June, and the population reached high abundance in the following 10 days (40 individuals per 10 sweeps). Although individuals were found along the ditch densely covered with grasses and scattered dicotyledons, *R. cuspidatus* was also aggregated on several plants of *Artemisia vulgaris* L. Only several *R. cuspidatus* specimens were swept in the adjacent sugar beet plot edge, while no individuals were caught within plot-2 or along other plot borders.

### ‘*Ca.* P. solani’ strains involved in epidemic RTD occurrence

**Figure 1:**
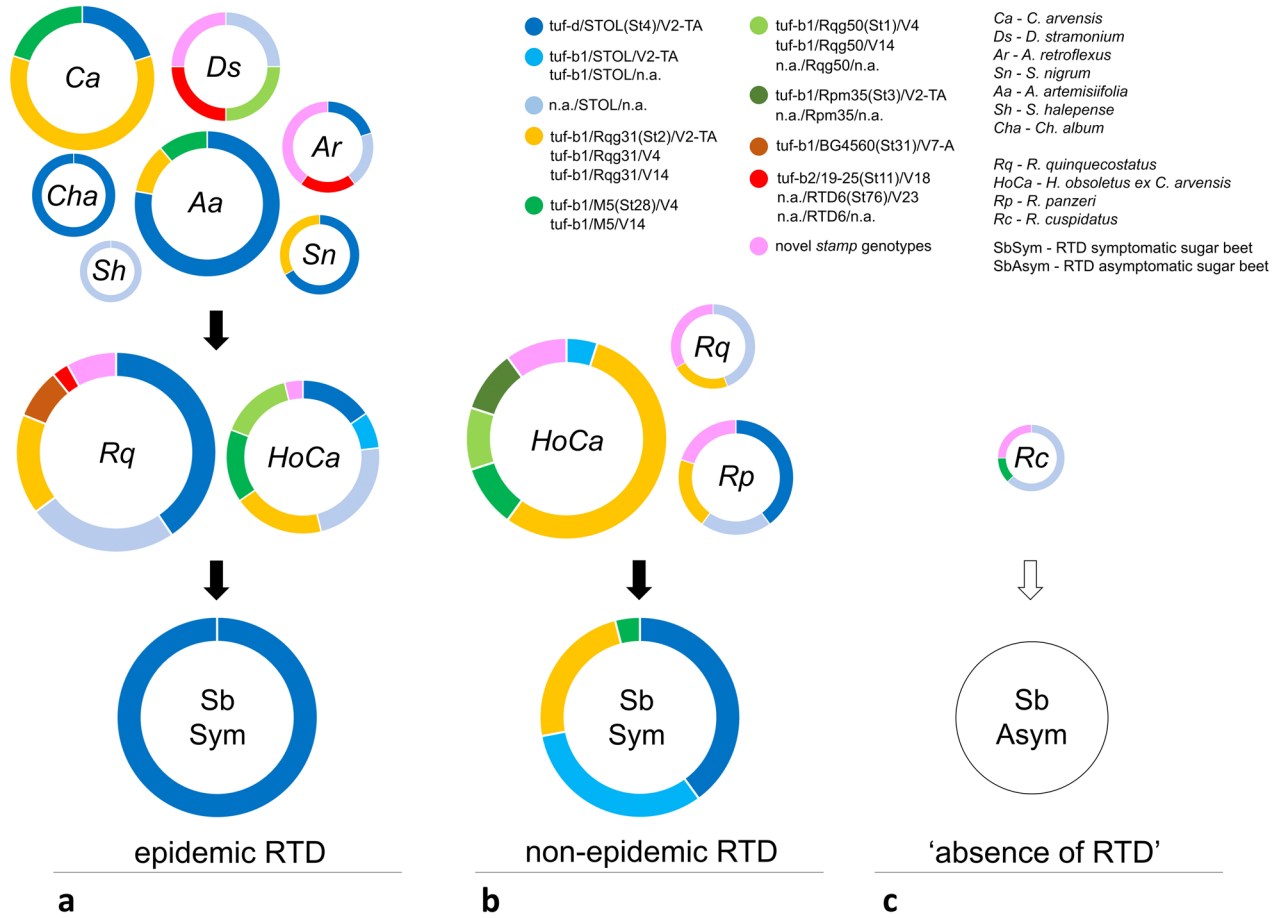
Genetic diversity of CaPsol strains involved in different sugar beet RTD occurrences in Rimski Šančevi: (**a**) epidemic RTD in plot-1 in 2020; (**b**) non-epidemic RTD in plot-1 in 2021; (**c**) ‘absence of RTD’ in plot-2 in 2021. CaPsol hosts, depicted as circle diagrams, are abbreviated as noted in the legend. The size of the circles corresponds to the percentage of CaPsol-positive samples in the total number of analyzed samples of a particular host. The colors of the diagrams represent CaPsol strains described in the legend, while the size of the colored part of the ring corresponds to the prevalence of a specific genotype. Details regarding novel *stamp* genotypes depicted in pink are provided in Tables 1 and 2, and S1.

**Figure 2:**
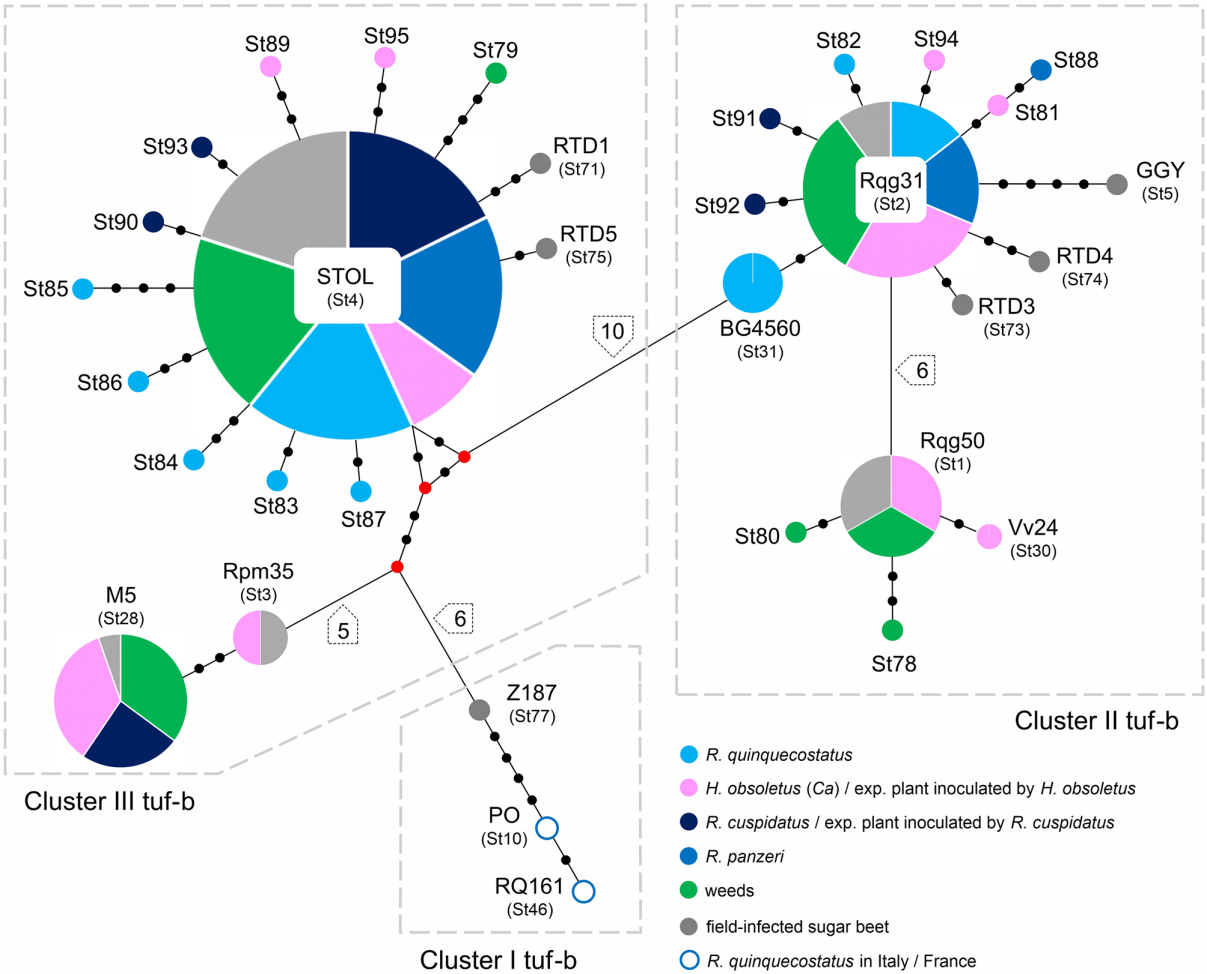
Median-Joining network of CaPsol *stamp* genotypes involved in RTD of sugar beet associated with tuf-b1 and tuf-d genotypes or with unknown *tuf* affiliation. Genotypes previously detected in RTD-affected sugar beet, as well as genotypes associated with *R. quinquecostatus* sensu Holzinger et al.^15^ in Italy and France, were included in the analysis ^4,16,29^. Circles correspond to a particular CaPsol *stamp* genotype. Circle sizes are proportional to the genotype frequency and colors correspond to the associated host as described in the legend. The black dots on the connecting lines represent the number of mutations, so as do the numbers noted in pentagons next to the connecting lines. The red dots in the network are median vectors representing missing or unsampled intermediate genotypes.

Sugar beet plot-1 was selected for tracing CaPsol strains in different hosts in 2020, because RTD occurred with epidemic severity in this plot in 2019. Populations of *R. quinquecostatus* and *H. obsoletus* (*Ca*) in plot-1 were both CaPsol-infected in 2020 (Table 2). The population of *R. quinquecostatus* had a high CaPsol infection rate of 67%, with more than half of the infected individuals bearing the STOL (St4) *stamp* genotype (25/38). Most of those strains (16/25) were genotyped as dSTOLg (tuf-d/STOL/V2-TA), while in the remaining 9 samples amplification of the *tuf* and *vmp1* genes was unsuccessful (Table 2; Fig. 1). Genotype Rqg31 (St2) was found in 6 individuals, while strain tuf-b1/BG4560(ST31)/V7-A was detected in three individuals. Three novel *stamp* sequence variants were also found, each per one individual: St82, St83 and St84 (Fig. 2). In addition, genotype RTD6 (St76), belonging to the tuf-a epidemiology, was detected in one *R. quinquecostatus* individual. CaPsol infection rate of *H. obsoletus* population was 42%, with less than half of the infected individuals having the STOL *stamp* genotype (12/26) (Table 2; Fig. 1). Four of them were infected with dSTOLg, two with strain tuf-b1/STOL/V2-TA, while the genotypes of six CaPsol strains remained uncharacterized on genes other than *stamp*. Most of the CaPsol strains infecting *H. obsoletus* belonged to *stamp* genotypes Rqg31, Rqg50 (St1) and M5 (St28), each present in an almost equal number of individuals, and mostly associated with the tuf-b1 genotype and *vmp1* profiles: V2-TA, V4 or V14. Another novel *stamp* sequence variant, St89, was detected in one individual (Fig. 2).

All weed species found to be CaPsol-infected in plot-1 were harboring the STOL *stamp* genotype. CaPsol strain dSTOLg was the only strain detected in *Ch. album* (3/3) and was prevalent in *A. artemisiifolia* (7/9) (Table 1; Fig. 1). It also infected *S. nigrum* and *A. retroflexus*. Only 3/15 CaPsol positive *C. arvensis* samples were infected with dSTOLg. The remaining CaPsol strains detected in this reservoir plant coincided with the genotype diversity found in the associated *H. obsoletus* population (Fig. 1). Inconsistency was observed for two genotypes; Rqg50 (4/26 *H. obsoletus* individuals) was not found in *C. arvensis* and the novel St89 (1/26 *H. obsoletus*) was not found in any of the tested plants. Three novel *stamp* sequence variants detected in weeds, St78, St79 and St80 (Fig. 2), were not found in any of the cixiids. CaPsol strains of the tuf-a epidemiology were found in tentative reservoirs: multilocus genotype tuf-b2/19-25(St11)/V18 in *D. stramonium* and RTD6(St76)/V23 in *A. retroflexus*.

After the 2020 RTD outbreak in plot-1 was evaluated as epidemic, genotyping of CaPsol strains from 25 RTD-symptomatic sugar beets revealed the presence of a single strain in all analyzed plants, dSTOLg (Fig. 1; Table S2).

### ‘*Ca.* P. solani’ strains involved in non-epidemic RTD occurrence

Compared to 2020, the population of *R. quinquecostatus* was severely reduced at the same microsite (NE boundary strip) in plot-1 in 2021, and present syntopically with *R. panzeri*. This resulted in the mixing of populations of these two congeneric species, which cannot be visually distinguished in the field. Both cixiids were CaPsol-infected and present in low numbers. The more abundant *R. quinquecostatus* population had a lower CaPsol infection rate (36%) than *R. panzeri* (42%) (Table 2). Almost half of the CaPsol-infected *R. quinquecostatus* individuals had the STOL (St4) *stamp* genotype (4/9), while Rqg31 (St2) was present in two individuals, and three novel *stamp* variants were also detected: St85, St86 and St87 (Fig. 2). CaPsol strain dSTOLg was found in *R. panzeri* (2/5). Multilocus genotype tuf-b1/Rqg31/V2-TA was detected in one *R. panzeri*, alongside another novel *stamp* genotype, St88 (Fig. 2). Though *H. obsoletus* (*Ca*) abundance in 2021 was the same as in 2020, its CaPsol infection rate was higher and reached 67% (20/30). Half of the *H. obsoletus* individuals were infected with Rqg31 (11/20), mainly genotypes tuf-b1/Rqg31/V2-TA and tuf-b1/Rqg31/V14 (Table 2). CaPsol genotype tuf-b1/Rpm35(St3)/V2-TA and two novel *stamp* variants, St94 and St95, were found only in the *H. obsoletus* population in this study. STOL genotype was detected in only 1/20 CaPsol-positive *H. obsoletus* in 2021 and was associated with the tuf-b1 genotype (Fig. 1).

The RTD severity in plot-1 was evaluated in 2021 as non-epidemic, because its assessment revealed a significant decrease in the number of symptomatic sugar beets compared to 2020, when this plot was affected by an epidemic RTD outbreak. A total of five CaPsol genotypes were found in 2021 RTD-symptomatic plants (Fig. 1; Table S2). CaPsol strain dSTOLg was present in less than half of the analyzed sugar beets (10/25) indicating a notable decrease in the incidence of this genotype, which was predominant in the same plot the previous year when epidemic RTD occurred. Prevalent CaPsol strains in 2021 were tuf-b1/STOL/V2-TA detected in 8/25 samples, and genotypes tuf-b1/Rqg31 and tuf-b1/M5, detected in 8 samples in total, and associated with the same *vmp1* profiles as the strains detected in *H. obsoletus* in the same year (Table S2).

### ‘*Ca.* P. solani’ strains persisting in the ‘absence of RTD’

RTD-free plot-2 was included in the research in 2021 for the ‘absence of RTD’ scenario. The only cixiid found on plot-2 was *R. cuspidatus* with a population infection rate of 23% (Table 2). The STOL *stamp* genotype dominated in *R. cuspidatus* individuals (5/8), while one individual had the M5 genotype, and two individuals were each infected with novel *stamp* variants, St90 and St91 (Fig. 2). No RTD symptoms were present in plot-2 in 2021 and further analysis of 15 asymptomatic sugar beets confirmed the absence of RTD on this plot.

### Genetic diversity and relatedness of the ‘*Ca.* P. solani’ strains involved in different RTD scenarios

CaPsol strains involved in all three RTD occurrence scenarios, except for strains with 19–25 and RTD6 *stamp* genotypes of tuf-a epidemiology, showed affiliation to tuf-b genetic Clusters II and III in the *stamp* genealogical network (Fig. 2). Most of the novel *stamp* sequence variants are grouped into Cluster III and are derived from the most frequently detected genotype, STOL (St4). The other 8 novel *stamp* genotypes were grouped into Cluster II tuf-b, as genetically related to the Rqg31 (St2) or Rqg50 (St1). Novel *stamp* genotypes were found in each of the assessed putative cixiid vectors and in two tentative reservoir plant species, while field sugar beets affected by RTD were all infected with previously known CaPsol strains. Each novel genotype was found per one insect or plant sample only, while genotypes St10 (strain PO) and ST46 (RQ161) from Cluster I, which were previously associated with *R. quinquecostatus* in Italy and France, were not found.

**Figure 3:**
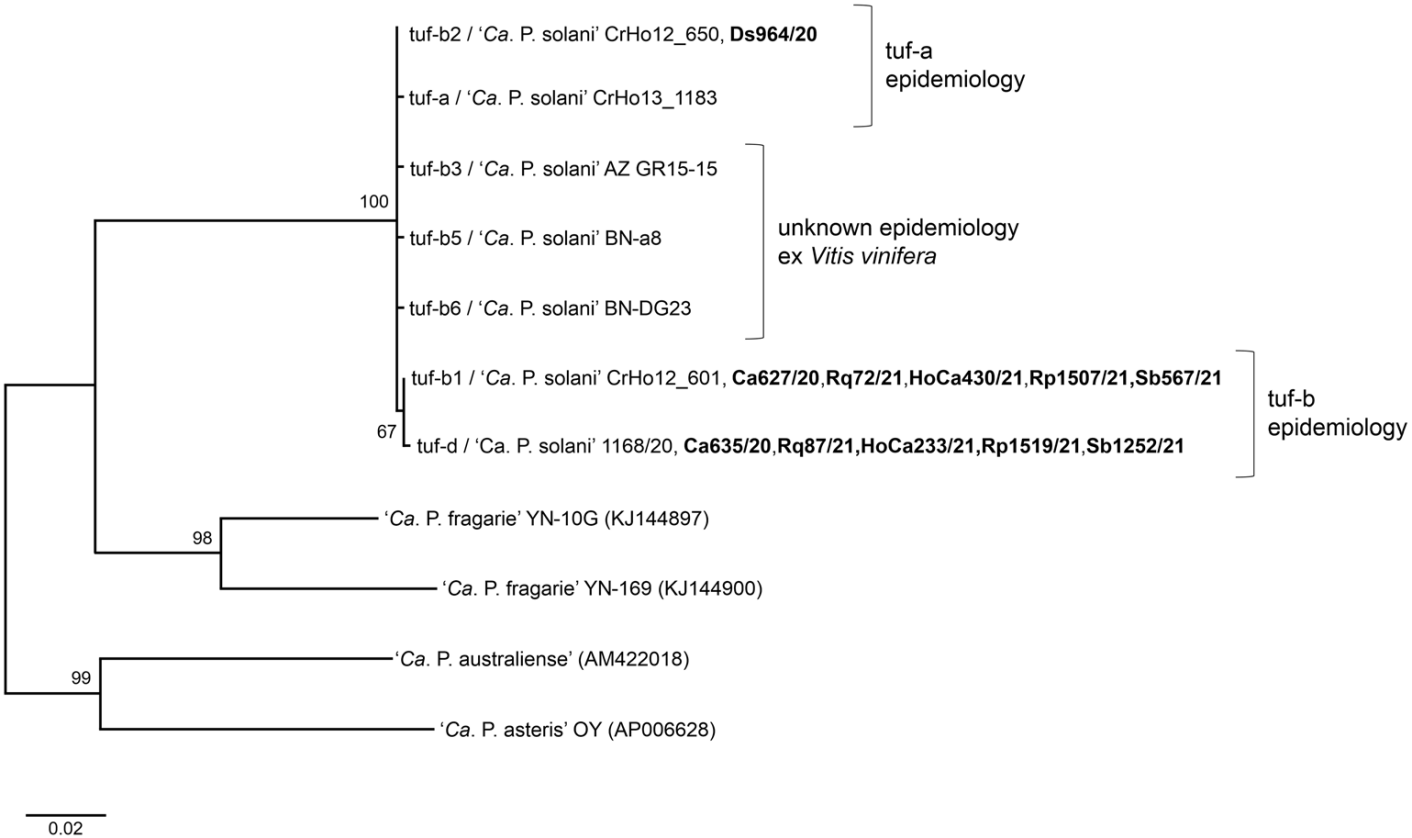
Maximum likelihood phylogenetic tree inferred from CaPsol *tuf* gene sequences of *tuf* genotypes reported in this and previous studies. A total of 7 published CaPsol *tuf* genotypes were included in analysis, while four *tuf* sequences of the other ‘*Candidatus* Phytoplasma’ species were used as outgroups. Strain names are provided for CaPsol sequences, while acc. numbers of outgroups are additionally noted in parentheses. Representative CaPsol *tuf* strains from this study, corresponding to the reference strain, are labeled in bold. Bootstrap support values are provided adjacent to branches.

Tuf-d type was found exclusively associated with the STOL *stamp* genotype, while tuf-b1 was mostly affiliated with other *stamp* genotypes (Rqg31, M5, Rqg50, Rpm35, BG4560, Vv24), occasionally associated with the STOL genotype and also found associated with one novel *stamp* sequence variant (St81; see Table S3b). Sequencing of the *tuf* gene showed that there were no additional mutations in strains of the tuf-b1, tuf-b2 and tuf-d genotypes, and that all analyzed samples share 100% identity with the reference strains (accession numbers provided in Tables 1 and 2). A maximum likelihood phylogenetic tree constructed from 7 published CaPsol *tuf* genotypes, of which three were detected in this study, and *tuf* sequences of other ‘*Candidatus* Phytoplasma’ species used as outgroups, clustered tuf-b1 and tuf-d strains into a well-supported secluded branch (Fig. 3). This finding, corroborated by the genetic clustering of *stamp* genotypes (Fig. 2), suggests that tuf-b1 and tuf-d CaPsol strains share the same tuf-b epidemiological cycle. However, the other genotypes, tuf-b3, b5 and b6 (identified in *Vitis vinifera* L.^25,26^), as well as tuf-a and b2 genotypes of the tuf-a epidemiology, showed ambiguous genetic relations (Fig. 3).

### ‘*Ca.* P. solani’ transmission trials and evaluation of RTD symptoms

#### Individual plant transmission trials

*Reptalus quinquecostatus* and *H. obsoletus* (*Ca*) from plot-1 were released in 2020 on 10 healthy test plants each: five periwinkles and five sugar beet seedlings. In 2021, trials with *R. quinquecostatus* could not be repeated because this cixiid was present in small numbers and syntopically with *R. panzeri*. However, tests with *H. obsoletus* were repeated on the same number of test plants and, in addition, *R. cuspidatus* from RTD-free plot-2 was enrolled in individual plant transmission trials.

**Figure 4:**
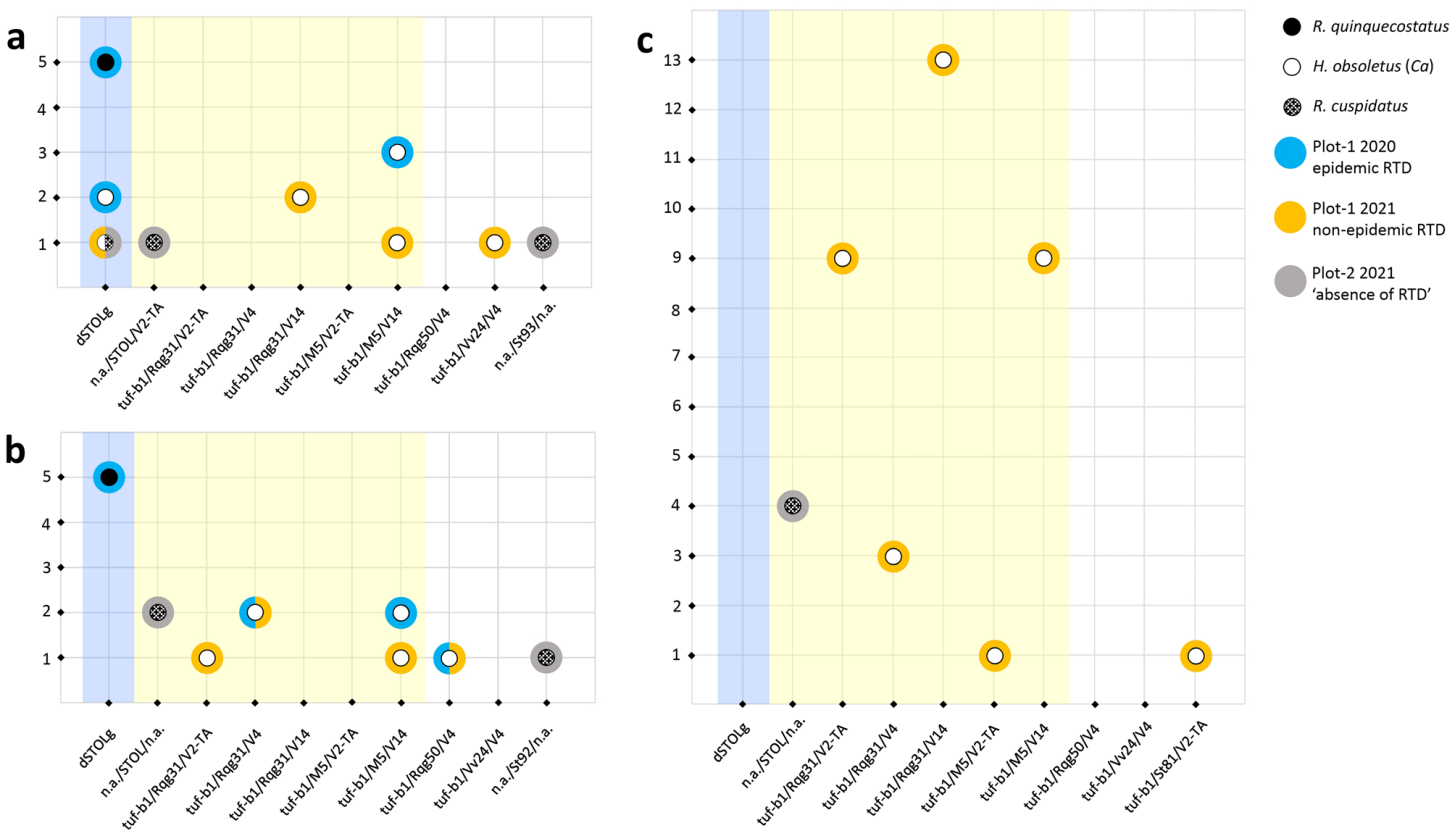
Scatter plot of the CaPsol strains transmitted to experimental plants by the three cixiid species: *R. quinquecostatus *sensu Holzinger et al*.*^15^*, H. obsoletus* (*Ca*) and *R. cuspidatus.* (**a**) individual sugar beets; (**b**) individual periwinkles; (**c**) sugar beets in semi-field cage experiments. CaPsol multilocus genotypes transmitted to experimental plants are labeled on the x-axis, while the number of infected test plants is labeled on the y-axis. Each of the three vectors is represented by a circle as described in the legend. A colored halos correspond to specific RTD severity, i.e., where and when vector populations were sampled. The blue background highlights dSTOLg, the CaPsol strain predominating in RTD-affected sugar beets in 2020 epidemic RTD occurrence, while the yellow encompasses CaPsol strains prevailing in RTD-affected sugar beets the 2021 non-epidemic disease occurrence.

At the end of July, 30 days after inoculation (30 DAI), all experimental sugar beets exposed to *R. quinquecostatus* (2020) and *H. obsoletus* (2020/2021) developed leaf RTD symptoms—yellowing of older leaves, with onset of necrosis (Fig. S2a). During the next two weeks, necrotic patches were spreading, leaves were drying, and whole plants began to wilt. Further decline of plants was more or less progressive and therefore some of them were sampled 50 DAI before their complete collapse (Fig. S2b). The remaining test sugar beets were sampled in the scheduled end of the experiment, 90 DAI, by which a variety of leaf symptoms was developing, mainly on older leaves, but occasionally also on younger ones (see various leaf RTD symptoms in Fig. S2c). None of the experimental sugar beets exhibited the most prominent RTD symptom, rubbery taproot. All inoculated periwinkles (5 by *R. quinquecostatus* and 10 by *H. obsoletus*) began to show symptoms of chlorosis and virescence 30 DAI and were sampled 90 DAI. Transmission trials with both cixiid species resulted in the successful CaPsol infection of all experimental plants. The *R. quinquecostatus* population transmitted only the dSTOLg strain to all test plants, sugar beets and periwinkles (Fig. 4a,b; Table S3a). *H. obsoletus* also transmitted dSTOLg to sugar beet, both in 2020 and in 2021, but only to 2/5 and 1/5 test plants, respectively. The CaPsol genotype most often transmitted to sugar beets by *H. obsoletus* was tuf-b1/M5/V14 (4/10), followed by tuf-b1/Rqg31/V14 (2/10). CaPsol strain tuf-b1/Vv24/V4 was detected for the first time in this study in experimental sugar beet inoculated by this vector. The diversity of CaPsol genotypes in periwinkles inoculated by *H. obsoletus* mostly matched the diversity of genotypes that its population transmitted to sugar beets (Fig. 4a,b; Table S3a).

None of the sugar beets exposed to *R. cuspidatus* developed RTD symptoms, nor did periwinkles express any of the typical phytoplasma-infection symptoms, and thus all plants were sampled 90 DAI as asymptomatic. Nevertheless, transmission trials with *R. cuspidatus* resulted in successful CaPsol-infection of 3/5 sugar beets and 3/5 periwinkles. Most of the transmitted CaPsol strains could not be genotyped on genes other than *stamp*, nevertheless, the STOL genotype was prevailing (Fig. 4a,b; Table S3a). The dSTOLg strain was transmitted by *R. cuspidatus*, along with two novel *stamp* variants: St93—detected in experimental sugar beet, and St92—found in periwinkle (Fig. 2; Fig. 4a,b; Table S3a).

#### Semi-field transmission trials

CaPsol trials to sugar beet, performed in net cages in semi-field conditions on sugar beet plot-2 (Fig. S3a), showed a drastic difference in the transmission capability of the tested *H. obsoletus* (*Ca*) and *R. cuspidatus* populations. Leaf symptoms of RTD started to appear 30 DAI in a cage with released *H. obsoletus*. By mid-August, 45 DAI, most of the symptomatic sugar beets began to decline, developing progressive necrosis and wilting. Before their complete decline, 32 sugar beets, heavily affected by RTD, were uprooted and sampled 50 DAI (Fig. S3b). All of these sugar beet plants had rubbery taproot. Of the eight plants remaining alive at 90 DAI, four had mild leaf symptoms, and four were asymptomatic, while none had rubbery taproot. All 32 sugar beets with typical RTD symptoms were CaPsol-infected, as were four sugar beets with mild leaf symptoms. The remaining four asymptomatic plants were CaPsol negative. As in individual plant tests, the CaPsol genotype tuf-b1/Rqg31 dominated in sugar beets inoculated in a cage with *H. obsoletus* (25/36), especially strains associated with V2-TA and V14 *vmp1* profiles (Fig. 4a,c; Table S3a,b). Ten sugar beets were infected with the tuf-b1/M5 genotype mainly associated with the V14 profile (9/10), which was also transmitted in individual plant trials (4/10 sugar beets, 3/10 periwinkles). One CaPsol genotype with a novel *stamp* variant, tuf-b1/St81/V2-TA, was transmitted to only one sugar beet plant (Figs. 2; 4c).

In the cage with released *R. cuspidatus*, all 40 sugar beets remained asymptomatic at 90 DAI. Though asymptomatic, 4/40 plants were CaPsol positive and infected with strains of the STOL *stamp* genotype, all uncharacterized on genes other than *stamp*, as recorded in individual plant trials (Fig. 4c; Table S3a,b). All 40 sugar beets from the control cage, which were not exposed to insects, were also asymptomatic 90 DAI (Fig. S3c) and tested negative for CaPsol (Table S3b).

## Discussion

Our previous research on RTD occurrence across the Pannonian Plain showed that the disease can significantly vary spatially^4^, suggesting that local epidemiological aspects play an important role in disease severity. In this epidemiological study, three RTD occurrence scenarios: epidemic, non-epidemic and ‘absence of RTD’, were successfully assessed in two growing seasons (2020–2021) in the sugar beet field in Rimski Šančevi, which proved to be an RTD-fluctuating experimental polygon. As a result, three CaPsol cixiid vectors were revealed in situ, each in a specific RTD scenario: (1) *R. quinquecostatus *sensu Holzinger et al*.*^15^, identified for the first time as a CaPsol vector to a crop plant (sugar beet), and proposed culpable for the 2020 RTD outbreak (epidemic scenario); (2) the prominent CaPsol vector, *H. obsoletus* (*Ca*), was identified as a threat to the sugar beet, adding it to the list of the crops targeted by this vector (non-epidemic scenario), while (3) *R. cuspidatus*, assessed in the ‘absence of RTD’ scenario, was experimentally confirmed as a novel CaPsol vector, though with a negligible role in RTD occurrence in Rimski Šančevi.

The population of *R. quinquecostatus* assessed during the 2020 RTD outbreak was highly infected with CaPsol (67%), which was higher than previously reported^10,14,16,29–31^. The prevalent CaPsol genotype in *R. quinquecostatus* was dSTOLg, the only strain found in field sugar beets affected in situ by the RTD epidemic outbreak. Transmission trials showed that dSTOLg was the only CaPsol strain that this cixiid transmitted to experimental plants, indicating that *R. quinquecostatus* could be the culpable vector for the RTD outbreak in 2020 in Rimski Šančevi. Predominance of particular CaPsol strains in epidemiological cycles associated with *R. quinquecostatus* (PO in Italy, RQ161 in France, dSTOLg genotype in Serbia) suggests that CaPsol outbreaks that share a certain epidemiological variable (vector in this case) can evolve independently in different micro-environments. CaPsol genotype STOL(St4)/V2-TA, previously found in *R. quinquecostatus* in Serbia, was associated with the tuf-b type, but its affiliation to either tuf-b1 or tuf-d genotypes remains unclear, as the *Hpa*II enzyme solely cannot differentiate the tuf-d type^3,10,14^. Although five tentative reservoir plants were found infected with tuf-d in Rimski Šančevi, which has also been recently reported in *C. foetida* and *Daucus carota* L. in Serbia^32^, the incidence and epidemiological relevance of the tuf-d type in CaPsol cycles of the tuf-b epidemiology remain to be evaluated. CaPsol strains other than dSTOLg were also detected in the assessed *R. panzeri* population, which makes its role in RTD currently unclear.

The involvement of *H. obsoletus* (*Ca*) in RTD was not surprising, as this cixiid is a prominent CaPsol vector in Serbia^8,10,12^. More importantly, it has been reported as a vector of CaPsol of the tuf-b type to sugar beet in France^18^. The incidence of dSTOLg was significantly lower in *H. obsoletus* than in *R. quinquecostatus*, and dSTOLg was less successfully transmitted to individual test plants by *H. obsoletus*. Moreover, in the semi-field cage test with *H. obsoletus*, none of the sugar beets were infected with dSTOLg. The most commonly transmitted CaPsol strains by *H. obsoletus* were tuf-b1/Rqg31/V4 and tuf-b1/Rqg31/V14, with the latter previously reported in the *H. obsoletus* (*Ca*) epidemiological cycle in Serbia^12^. Regardless of RTD severity, diversity of CaPsol strains transmitted by *H. obsoletus* changed very little. In 2021, *H. obsoletus* was the predominant cixiid in plot-1 with a high infection rate (67%), yet RTD did not occur with epidemic severity. Though its high efficiency in transmitting CaPsol was confirmed, molecular typing suggested that *H. obsoletus* was not culpable for the dSTOLg outbreak in sugar beet plot-1 in 2020. However, a sugar beet field in Bačko Gradište (Serbia), evaluated in 2020 as a locality with epidemic RTD occurrence, had minor presence of the dSTOLg strain (5/15 CaPsol-infected sugar beets)^4^. This finding suggests that other CaPsol strain(s) can contribute to RTD epidemic severity and that other vectors, such as *H. obsoletus*, could be involved in the RTD outbreak. The minor role of *H. obsoletus* (*Ca*), or its non-participation in CaPsol outbreaks, was previously reported or suggested for other crops^13,14,16,29,31^. Our results, compiling molecular epidemiology and transmission tests, show that *R. quinquecostatus* can be a prominent CaPsol vector, even if present in sympatry with another vector, thus upgrading the relevance of *R. quinquecostatus* from a vector possibly responsible for alternative CaPsol epidemiological cycles, to a cixiid solely culpable for disease outbreaks.

Although the tested *R. cuspidatus* population was active near (in) sugar beet plot-2 with a two-year ‘absence of RTD’, transmission trials revealed that *R. cuspidatus* is a CaPsol vector capable of infecting periwinkle and sugar beet plants. The tested *R. cuspidatus* population showed lower transmission capacity compared to *R. quinquecostatus* and *H. obsoletus*, especially in semi-field cage experiments where only 10% of sugar beet was CaPsol-infected. Considering that sugar beet experimentally infected with CaPsol in France showed disease symptoms 5 months after inoculation^18^, the lack of observable symptoms in our study may be a consequence of early uprooting, performed 90 DAI (3 months). *Reptalus cuspidatus* was previously reported as CaPsol-infected and suspected to be involved in “bois noir”^33,34^. The preference of its adults for *A. vulgaris* was reported also in Italy, where the plant was found to be CaPsol positive^34,35^. Albeit the CaPsol infection rate of *R. cuspidatus* population in Rimski Šančevi was 24% and thus higher than previously reported^34^, its role in RTD in Rimski Šančevi appears minor to negligible, while involvement in other CaPsol epidemiological cycles remains to be evaluated. All CaPsol strains infecting *R. cuspidatus*, or transmitted to experimental plants, belonged to the tuf-b epidemiology. Strains associated with the tuf-b2 genotype, previously found to infect *R. cuspidatus* in Switzerland^34^, were not detected in our study.

The presence of the RTD6 (St76) *stamp* genotype in *R. quinquecostatus*, which belongs to the tuf-a epidemiology, corroborates previous findings^16,31^. Unlike the matching occurrence of St6 *stamp* genotype (associated with *U. dioica*) in *R. quinquecostatus* and grapevines in France^16^, none of the analyzed sugar beets from plot-1 were infected with strains of the tuf-a epidemiology, although the RTD6 genotype was in situ infecting *R. quinquecostatus* population. However, RTD6 was found in a tentative reservoir plant (*A. retroflexus*) in plot-1 and was previously recorded in sugar beet in other localities where non-epidemic RTD occurred^4^. CaPsol strains of the tuf-a epidemiology were rarely found in RTD-affected sugar beet throughout the Pannonian Plain^4^, while *H. obsoletus ex U. dioica* was previously found ineffective in CaPsol transmissions to sugar beet^18^.

The genes targeted in the genotyping of CaPsol strains involved in RTD in Rimski Šančevi, provided reliable insight into disease epidemiology. Inconsistent amplification of *tuf* and *vmp1* genes did not substantially disrupt the reconstruction of CaPsol pathways, though this phenomenon was previously reported^4,29,36,37^. The most prominent RTD symptom, rubbery taproot, was not developed by any sugar beet infected in the individual plant inoculation tests, likely because of the regular watering regime. On the contrary, sugar beets that were inoculated by *H. obsoletus* in cage experiments and later exhibited the characteristic rubbery symptoms, were exposed to environmental conditions including low amounts of precipitation, averaging 221 mm over a five-month period (May–September 2021). The cixiid planthopper *P. leporinus* was not detected in Rimski Šančevi, nor were nests or nymphs of any cixiid species found in the sugar beet rhizosphere during the sampling. The sympatric occurrence of *R. quinquecostatus*, *R. panzeri*, *R. cuspidatus* and *H. obsoletus* (*Ca*) was observed in 2021 on field margins and within sugar beet crops in other locations in northern Serbia (unpublished data). Since *R. quinquecostatus* can be found in different crops^10,13,14^, its populations are most likely associated with plant(s) of the spontaneous vegetation growing near or inside arable land, rather than with cultivated plants. Agroecosystems provide novel biotic environment for vectors. In addition, nitrogen-based fertilization practices attract Auchenorrhyncha species^38^, leading to aggregation of vector populations on crop plants, weeds and ruderal vegetation. Eventually, vectors can adapt to crops, as recorded for *R. panzeri* and *P. leporinus*^13,39^.

Plant diseases caused by CaPsol often manifest recurrent epidemics. After a CaPsol outbreak, that may cause significant yield losses, the disease enters a restraint phase when it is suppressed and, in the context of agroeconomic losses, irrelevant. Temporal discrepancy in the identification of CaPsol vectors and reservoir plants, and evaluation of the damage caused by CaPsol to the crop, is the main obstacle in predicting disease occurrence and severity. Targeting key vectors, their host plants, and CaPsol inoculum reservoirs is currently the most effective precautionary measure that can be implemented during anticipation of disease occurrence.

## Methods

### Experimental sugar beet field

This study of RTD epidemiology was conducted from April to October in two consecutive years, 2020 and 2021, at the experimental sugar beet field of the Institute of Field and Vegetable Crops in Rimski Šančevi (Novi Sad, Vojvodina, north Serbia), i.e., two sugar beet plots 1.2 km apart: plot-1 of 5 ha and plot-2 of 2 ha. Sugar beet of the certified hybrid “Original” was sown and maintained as previously described^3^.

All methods applied in this study, including sampling, complied with Serbian guidelines and legislation. Access to the experimental field was granted and permission to collect samples was obtained from the Institute of Field and Vegetable Crops in Novi Sad.

### Sampling of sugar beets and tentative ‘*Ca.* P. solani’ reservoir plants

Both sugar beet plots were inspected in mid-September 2020 and 2021 for the presence of RTD symptoms. The severity of RTD occurrence was assessed according to previously described methodology^4^. Twenty-five RTD-symptomatic sugar beets were sampled from plot-1 in each year. Fifteen previously reported RTD-symptomatic sugar beets from plot-1 sampled in 2020^4^ were supplemented for this study with an additional 10 symptomatic plants from the same year and plot. As RTD symptoms were not present on plot-2, 15 asymptomatic sugar beets were collected each year. All of the sampled plants had no signs or symptoms of any other root pathogen or pest. Half a gram of taproot tissue was sampled for further analysis. The inventory of tentative reservoir plants in plot-1 was done in June 2020. Symptoms of phytoplasma infection on weeds were monitored until mid-September when sampling of the leaves (half a gram) was performed. The number of collected samples corresponded to the abundance of a particular weed. All sugar beet root and weed leaf samples were stored at − 20 °C until further DNA isolation.

### Sampling and identification of putative ‘*Ca.* P. solani’ cixiid vectors

The presence of cixiids was monitored from mid-May to mid-August in 2020 and 2021 in sugar beet plot-1, while plot-2 was inspected only in 2021. Insects were usually collected once a week, or more frequently in June and July, using an entomological net and a mouth aspirator. Sweeping was done inside the crop and along the boundary strips. Collected insects were stored in 96% ethanol and identified under a Leica S9E stereomicroscope by external morphology and male genitalia^15^.

*Reptalus quinquecostatus* has recently undergone a taxonomic revision^40^. This cixiid was thoroughly researched across Europe under the name ‘*R. quinquecostatus’* as a highly potent CaPsol vector presumably involved in several plant diseases^10,14,16,29,31^. To provide traceability to published data regarding this species, we referred to it as *R. quinquecostatus *sensu Holzinger et al*.*^15^ in our research. The identity of *R. quinquecostatus* and *R. panzeri* males was determined by morphological difference in the anal tube, i.e., by the presence of a distinct process with a left orientation in *R. quinquecostatus*, which is absent in *R. panzeri*. The female specimens of these two congeneric species could not be reliably distinguished, hence they were subjected to molecular identification. Internal transcribed spacer 2 (ITS2) of the ribosomal DNA was amplified using primer pair ITS2fw/ITS2rv in a 25-μL final reaction volume containing 2 μl of template DNA (isolated as described below), 1 × PCR Master Mix (Thermo Scientific, Vilnius, Lithuania) and 0.4 µM of each primer under the thermal conditions as previously described^41,42^. As *R. cuspidatus* adults bear platellae on the first segment of the hind tarsus, unlike the other two *Reptalus* species^15^, RFLP digestion of ITS2fw/ITS2rv amplicons with *Taq*I enzyme was omitted from further analysis. Thus, the affiliation of the females to either *R. quinquecostatus* or *R. panzeri* was determined only based on differences in the length of ITS2 amplicons obtained in PCR^42^.

### Isolation of plant and insect DNA and ‘*Ca.* P. solani’ detection

DNA isolation from field-collected and experimental plants was done following the CTAB protocol^43^. Genomic DNA from individual insects was isolated using a modified CTAB method^17^. As the applied extraction procedure involves the homogenization of insects, aedeagi of 10 males per species/population were previously removed and stored in glycerol at 4 °C. Isolated DNA was kept at − 20 °C until further analysis. CaPsol detection in plants and insects was based on the amplification of CaPsol-specific *stamp* gene in nested PCR with StampF/R0 and StampF1/R1 primers^44^. CaPsol strain 429/19 was used as a positive control^3^. The obtained PCR amplicons were separated in 1% agarose gels, stained with ethidium bromide and visualized under UV light.

### Real-time PCR detection of ‘*Ca*. A. phytopathogenicus’ in field-collected sugar beets

Presence of ‘*Ca*. A. phytopathogenicus’ was verified using the TaqMan real-time PCR protocol targeting the *hsp20* gene (heat shock protein 20) of ‘*Ca*. A. phytopathogenicus’^45^. Final reaction volumes of 15 μL contained 1 × TaqMan qPCR Master Mix (Nippon Genetics Europe), 1 μL template DNA, 0.2 μL Uracil-N-Glycosylase (UNG), 450 nM of each primer and 200 nM probe. The qPCR was performed in a Magnetic Induction Cycler, Mic (Bio Molecular Systems, Australia) with cycling parameters as previously described^45^. Each reaction included a DNA-free blank assay, a negative control corresponding to an RTD-asymptomatic sugar beet, and positive control of ‘*Ca*. A. phytopathogenicus’, strain HN1220/5^3^. Additional qPCR assay targeting the *nad5* gene (encoding NADH-ubiquinone oxidoreductase chain 5) of *Beta vulgaris* L., using the same positive control (HN1220/5), was separately performed to confirm the presence and evaluate the quality of the DNA template^45^. Data analysis was performed using micPCR^©^ software Version 2.6.4 (Bio Molecular Systems, Australia).

### Multilocus genotyping of ‘*Ca*. P. solani’ strains

All CaPsol strains from field-collected sugar beets, weeds, insects and experimental plants, were subjected to molecular typing on three genes: *stamp*, *tuf* and *vmp1*. The final volume of the PCR mix for each reaction contained 1 ul of the diluted (1:50) extracted DNA for plant material or 2ul of the isolated insect DNA, 1 × PCR Master Mix and 0.4 μM of each primer. Each of the three genes was amplified in the corresponding nested reaction using 1 μL of direct PCR product diluted 5 × in sterile water.

Nested s*tamp* gene sequences were commercially obtained from Macrogen Inc. (Seoul, South Korea), edited using FinchTV v. 1.4.0 (http://www.geospiza.com) and aligned with CaPsol reference strains using ClustalX integrated into MEGA 5 software^46^. Accession numbers of the 18 novel *stamp* genotypes/sequence variants deposited in GenBank are provided in Tables 1 and 2, and Table S1. Specific “St” codes were assigned to *stamp* genotypes previously reported as involved in RTD^4^ and genotypes described in this study, continuing the *stamp* sequence variant list^28,29^ (Table S1). The genetic relatedness of CaPsol strains, based on the *stamp* gene, was assessed by a phylogenetic Median-Joining network constructed with NETWORK version 10.2 (www.fluxus-engineering.com)^47^. The applied settings had a default value of 0 for the ε parameter and maximum parsimony post-processing to obtain a network containing all the shortest trees. CaPsol strains belonging to the tuf-b epidemiology, reported previously^4^ and in this study as involved in RTD, along with strains PO (St10) and RQ161 (St46) detected in *R. quinquecostatus* in Italy and France^16,29^, were subjected to M-J analysis. The *tuf* gene was amplified by combining two PCR procedures, depending on the amplification efficacy: a nested protocol with primer pairs fTuf1/rTuf1 and fTufAY/rTufAY^21,48^ and a semi-nested protocol with fusAF1/tufBR1 and fusAF2/tufBR1 primers^49^. Further identification of the *tuf* type/genotype of the analysed CaPsol samples was done as previously described^4^. The *tuf* gene of representative CaPsol strains of each multilocus genotype found in each host was sequenced to verify the presence of additional genetic variability within the detected tuf-b and tuf-d types. A total of 28 representative *tuf* sequences equally distributed across genotypes and hosts were deposited in GenBank (acc. no. OP231764-OP231791). A Maximum likelihood tree was generated in MEGA X software by applying the Tamura 3-parameter model using a discrete Gamma distribution as the best-fit substitution model^50^. Sequences of the *tuf* gene of three other ‘*Candidatus* Phytoplasma’ species were used as outgroups: ‘*Ca*. P. fragariae’, ‘*Ca*. P. australiense’ and ‘*Ca*. P. asteris’. One thousand bootstrap replicates were performed to estimate the statistical significance of the inferred clades. Amplification of the *vmp1* gene was performed in nested PCR assays using primers StolH10F1/R1 and TYPH10F/R^36,51^. Identification of the *vmp1* profiles of the analysed CaPsol samples was done as previously described^4^.

### Experimental ‘*Ca*. P. solani’ transmission trials via putative cixiid vectors

Transmission trials of CaPsol to healthy sugar beet and periwinkle plants via naturally infected populations of putative cixiid vectors were performed in two experimental setups: (1) individual plant trials carried out in 2020 and 2021, and (2) semi-field trials performed in 2021 on sugar beet plot-2. In both experiments, the certified sugar beet hybrid “Original’’ was used. Plants for individual plant tests were seed-grown in mid-April, planted in pathogen-free soil and maintained in a climate chamber at 24 ± 1 °C (16/8 h light/dark period). Three cixiid species were enrolled in the trials after the CaPsol infection of insects was verified based on the analysis of a representative number of individuals. Populations of *R. quinquecostatus* and *H. obsoletus* (*Ca*) from plot-1 were tested in 2020 in individual plant experiments. In 2021, tests with *R. quinquecostatus* could not be repeated, as it was present in small numbers and syntopically with *R. panzeri*. However, trials with *H. obsoletus* were repeated, and in addition, *R. cuspidatus* from plot-2 was included in individual plant trials in 2021. Insects were collected at the end of June and released on individual experimental plants that were previously covered with plastic cylinders, with ventilation provided. Five sugar beets and five periwinkles were used in testing each of the putative vectors per year. Each experimental plant hosted 30 individuals of a certain species for 72 h. Insects were afterwards collected and stored in 96% ethanol. Experimental sugar beets were further maintained outdoors in a field net cage, while periwinkle plants were kept in a climate chamber. Five control sugar beets and periwinkles not exposed to insects were included in the experiment as negative controls. All plants were monitored daily for the development of symptoms and were sampled 90 DAI. Plants that had begun to decline earlier were sampled accordingly.

Semi-field CaPsol transmission trials to sugar beet were set up in 2021 in the plot-2 that was evaluated as RTD-free in 2020. Net cages (2 × 2 × 2.5 m) were installed on May 15th, covering 40 plants each that were subjected to the same agrotechnical protocol as the rest of the plot. Populations of *H. obsoletus* (*Ca*) from plot-1 and *R. cuspidatus* caught on ruderal vegetation in a ditch bordering plot-2, were enrolled in trials. A total of 250 individuals of each species were sampled at the end of June and were released each into a secluded cage. An additional cage without insects was included in the experiment as a negative control. Sugar beets in cages were visually evaluated for the development of leaf symptoms once a week or more frequently, depending on symptom severity and plant decline. Sampling of the sugar beets was done in the beginning of October, while declining plants were sampled accordingly.

## Supplementary Information

Supplementary Information.

## Data Availability

All data generated or analyzed during this study are included in this published article (and its Supplementary Information files).
