# Supplementary material for: Epidemiological role of novel and already known ‘Ca. P. solani’ cixiid vectors in rubbery taproot disease of sugar beet in Serbia
Source: Sci Rep. 2023 Jan 25;13:1433. doi: 10.1038/s41598-023-28562-8 (PMC9877035; doi:10.1038/s41598-023-28562-8)

Epidemiological role of novel and already known ‘*Ca*. P. solani’ cixiid vectors in rubbery taproot disease of sugar beet in Serbia

Andrea Kosovac, Živko Ćurčić, Jelena Stepanović, Emil Rekanović, and Bojan Duduk

Supplementary Table S1. List of CaPsol s*tamp* genotypes/sequence variants found to be involved in RTD of sugar beet in this and previous studies (Ćurčić *et al.*[1]; Ćurčić *et al.*[2]).

For each *stamp* genotype involved in RTD corresponding “St” sequence variant code is given according to Pierro *et al.*[3] (St1-St59 *stamp* sequence variants) and Quaglino *et al.*[4] (St60-St70 *stamp* sequence variants). “St” sequence variant codes are assigned to the novel *stamp* genotypes reported in Ćurčić *et al.*[2] (St71-St77) and for the novel *stamp* genotypes described in this research (St78-St95).

1. Ćurčić, Ž. *et al*. Rubbery taproot disease of sugar beet in Serbia associated with ‘*Candidatus* Phytoplasma solani’. *Plant Dis.* **105,** 255-263 (2021).
2. Ćurčić, Ž. *et al*. Multilocus genotyping of ‘*Candidatus* Phytoplasma solani’ associated with rubbery taproot disease of sugar beet in the Pannonian Plain. *Microorganisms* **9**, 1950; 10.3390/microorganisms9091950 (2021).
3. Pierro, R. *et al*. Proposal of a new Bois noir epidemiological pattern related to ‘*Candidatus* Phytoplasma solani’ strains characterized by a possible moderate virulence in Tuscany. *Pathogens*, **9,** 268; 10.3390/pathogens9040268 (2020).
4. Quaglino, F. *et al*. Molecular and spatial analyses reveal new insights on Bois noir epidemiology in Franciacorta vineyards. *Ann. Appl. Biol.* **179,** 151-168 (2021).

For each *stamp* genotype/sequence variant, the corresponding *tuf* genotype (determined in this or previous research regarding RTD) is given, along with belonging to a particular *stamp* tuf cluster.

Stamp genotype/strain (acc. no. reference strain)	*Stamp* “St” sequence variant	*Stamp* cluster affiliation	*Tuf* genotype	Host
Rqg50 (KC703019)	St1	Cluster II tuf-b	tuf-b1	*H. obsoletus* (*Ca*) ***<br>Field sugar beet *, **
			n.a.	*D. stramonium* ***<br>*H. obsoletus* (*Ca*) ***
Rqg31 (KC703017)	St2	Cluster II tuf-b	tuf-b1	*C. arvensis* ***<br>*S. nigrum* ***<br>*A. artemisiifolia* ***<br>*H. obsoletus* (*Ca*) ***<br>*R. panzeri* ***<br>Field sugar beet **, ***
			n.a.	*R. quinquecostatus* ***<br>*H. obsoletus* (*Ca*) ***
Rpm35 (KC703015)	St3	Cluster III tuf-b	tuf-b1	*H. obsoletus* (*Ca*) ***<br>Field sugar beet **
			n.a.	*H. obsoletus* (*Ca*) ***
STOL (FN813261)	St4	Cluster III tuf-b	tuf-b1	*H. obsoletus* (*Ca*) ***<br>Field sugar beet **, ***
			tuf-d	*C. arvensis* ***<br>*A. retroflexus* ***<br>*Ch. album* ***<br>*S. nigrum* ***<br>*A. artemisiifolia* ***<br>*R. quinquecostatus* ***<br>*H. obsoletus* (*Ca*) ***<br>*R. panzeri* ***<br>Field sugar beet *, **, ***

			n.a.	*A. retroflexus* ***<br>*D. stramonium* ***<br>*S. halepense* ***<br>*R. quinquecostatus* ***<br>*H. obsoletus* (*Ca*) ***<br>*R. panzeri* ***<br>*R. cuspidatus* ***
GGY<br>(FN813256)	St5	Cluster II tuf-b	tuf-b1	Field sugar beet **
SB5<br>(FN813266)	St8	Epidemiology tuf-a	tuf-a	Field sugar beet **
19-25<br>(FN813267)	St11	Epidemiology tuf-a	tuf-b2	*D. stramonium* ***
M5<br>(KP337316)	St28	Cluster III tuf-b	tuf-b1	*C. arvensis* ***<br>*A. artemisiifolia* ***<br>*H. obsoletus* (*Ca*) ***<br>Field sugar beet *, **, ***
			n.a.	*R. cuspidatus* ***
Vv24<br>(KC703022)	St30	Cluster II tuf-b	tuf-b1	Sugar beet inoculated by *H. obsoletus* (*Ca*) ***
BG4560<br>(FN813252)	St31	Cluster II tuf-b	tuf-b1	*R. quinquecostatus* ***
RTD1 / strain 1177/20<br>(MZ604948)	St71	Cluster III tuf-b	n.a.	Field sugar beet **
RTD2 / strain 1198/20<br>(MZ604952)	St72	Epidemiology tuf-a	n.a.	Field sugar beet **
RTD3 / strain 1443/20<br>(MZ604956)	St73	Cluster II tuf-b	n.a.	Field sugar beet **
RTD4 / strain 1447/20<br>(MZ604957)	St74	Cluster II tuf-b	n.a.	Field sugar beet **
RTD5 / strain 1453/20<br>(MZ604958)	St75	Cluster III tuf-b	n.a.	Field sugar beet **
RTD6 / strain 1562/20<br>(MZ604970)	St76	Epidemiology tuf-a	tuf-b2	Field sugar beet **
			n.a.	*A. retroflexus* ***<br>*R. quinquecostatus* ***
Z187<br>(MZ604974)	St77	Cluster I tuf-b	n.a.	Field sugar beet **
Ds958/20<br>(OP156882)	St78	Cluster II tuf-b	n.a.	*D. stramonium* ***
Ar1055/20<br>(OP156883)	St79	Cluster III tuf-b	n.a.	*A. retroflexus* ***
Ar1059/20<br>(OP156884)	St80	Cluster II tuf-b	n.a.	*A. retroflexus* ***
SbHo713/21<br>(OP156885)	St81	Cluster II tuf-b	tuf-b1	Sugar beet inoculated by *H. obsoletus* (*Ca*) ***
Rq104/21<br>(OP156886)	St82	Cluster II tuf-b	n.a.	*R. quinquecostatus* ***
Rq403/21<br>(OP156887)	St83	Cluster III tuf-b	n.a.	*R. quinquecostatus* ***
Rq423/21<br>(OP156888)	St84	Cluster III tuf-b	n.a.	*R. quinquecostatus* ***

Rq1497/21 (OP156889)	St85	Cluster III tuf-b	n.a.	*R. quinquecostatus* ***
Rq1498/21 (OP156890)	St86	Cluster III tuf-b	n.a.	*R. quinquecostatus* ***
Rq1511/21 (OP156891)	St87	Cluster III tuf-b	n.a.	*R. quinquecostatus* ***
Rp1531/21 (OP156892)	St88	Cluster II tuf-b	n.a.	*R. panzeri* ***
HoCa238/21 (OP156893)	St89	Cluster III tuf-b	n.a.	*H. obsoletus* (*Ca*) ***
Rc1451/21 (OP156894)	St90	Cluster III tuf-b	n.a.	*R. cuspidatus* ***
Rc1470/21 (OP156895)	St91	Cluster II tuf-b	n.a.	*R. cuspidatus* ***
WRc992/21 (OP156896)	St92	Cluster II tuf-b	n.a.	Periwinkle inoculated by *R. cuspidatus* ***
SbRc1276/21 (OP156897)	St93	Cluster III tuf-b	n.a.	Sugar beet inoculated by *R. cuspidatus* ***
HoCa90/22 (OP156898)	St94	Cluster II tuf-b	n.a.	*H. obsoletus* (*Ca*) ***
HoCa99/22 (OP156899)	St95	Cluster III tuf-b	n.a.	*H. obsoletus* (*Ca*) ***

* Ćurčić, Ž. *et al*. Rubbery taproot disease of sugar beet in Serbia associated with '*Candidatus* Phytoplasma solani'. *Plant Dis.* **105,** 255-263 (2021).

** Ćurčić, Ž. *et al*. Multilocus genotyping of '*Candidatus* Phytoplasma solani' associated with rubbery taproot disease of sugar beet in the Pannonian Plain. *Microorganisms* 9, 1950; 10.3390/microorganisms9091950 (2021).

*** found in this study

Supplementary Table S2. '*Ca*. P. solani' multilocus genotypes detected in field-collected sugar beets in plot-1 in two consecutive years, 2020 and 2021, during epidemic and non-epidemic RTD occurrence, respectively.

Year / epidemic or non-epidemic RTD	CaPsol positive/ total number of samples	CaPsol multilocus genotype (no. of samples)		CaPsol strain	GenBank acc. no. of *tuf* sequence
2020 / epidemic	25/25	tuf-d/STOL/V2-TA	(25)		
2021 / non-epidemic	25/25	tuf-d/STOL/V2-TA	(10)	Sb1252/21	OP231788
		tuf-b1/STOL/V2-TA	(8)	Sb567/21	OP231787
		tuf-b1/Rqg31/V2-TA	(2)		
		tuf-b1/Rqg31/V14	(4)	Sb564/21	OP231785
		tuf-b1/M5/V14	(1)	Sb565/21	OP231786

Supplementary Table S3. '*Ca*. P. solani' multilocus genotypes detected in experimentally inoculated sugar beet and periwinkle plants.

Table S3a. CaPsol multilocus genotypes detected in test plants from the individual plant tests.

Cixiid vector	Year	Experimental plants			
		Sugar beet		Periwinkle	
		CPs+ / No. of plants	CaPsol multilocus genotype (no. of samples)	CPs+ / No. of plants	CaPsol multilocus genotype (no. of samples)
R. quinquecostatus	2020	5/5	tuf-d/STOL/V2-TA (5)	5/5	tuf-d/STOL/V2-TA (5)
	2021	/	/	/	/
H. obsoletus<br>*ex C. arvensis*	2020	5/5	tuf-d/STOL/V2-TA (2)<br>tuf-b1/M5/V14 (3)	5/5	tuf-b1/Rqg31/V14 (2)<br>tuf-b1/M5/V14 (2)<br>tuf-b1/Rqg50/V4 (1)
	2021	5/5	tuf-d/STOL/V2-TA (1)<br>tuf-b1/Rqg31/V14 (2)<br>tuf-b1/M5/V14 (1)<br>tuf-b1*/Vv24/V4 (1)	5/5	tuf-b1/Rqg31/V2-TA (1)<br>tuf-b1/Rqg31/V14 (2)<br>tuf-b1/M5/V14 (1)<br>tuf-b1/Rqg50/V4 (1)
R. cuspidatus	2020	/	/	/	/
	2021	3/5	tuf-d**/STOL/V2-TA (1)<br>n.a./STOL/V2-TA (1)<br>n.a./**St93**/n.a (1)	3/5	n.a./STOL/n.a. (2)<br>n.a./**St92**/n.a. (1)

* *tuf* sequence GenBank acc no. OP231789, strain SbHo1279/21
** *tuf* sequence GenBank acc no. OP231791, strain SbRc1336/21

Table S3b. CaPsol multilocus genotypes detected in sugar beets from the semi-field cage experiments.

Cixiid vector	CaPsol positive/number of samples	CaPsol multilocus genotype (no. of samples)	
H. obsoletus<br>*ex C. arvensis*	36/40	tuf-b1/Rqg31/V2-TA<br>tuf-b1/Rqg31/V4<br>tuf-b1/Rqg31/V14<br>tuf-b1/M5/V2-TA<br>tuf-b1/M5/V14<br>tuf-b1*/**St81**/V2-TA	(9)<br>(3)<br>(13)<br>(1)<br>(9)<br>(1)
R. cuspidatus	4/40	n.a./STOL/n.a.	(4)
Negative control cage	0/40	/	/

* *tuf* sequence GenBank acc no. OP231790, strain SbHo713/21

Supplementary Figure S1. Epidemic RTD occurrence in experimental sugar beet plot-1 in 2020. Caption of the NE plot section heavily affected by RTD, photographed in early October 2020. The adjacent boundary strip and captured plot section hosted dense patch of weeds and aggregated *R. quinquecostatus* (sensu Holzinger *et al.* 2003) population earlier in the same year.

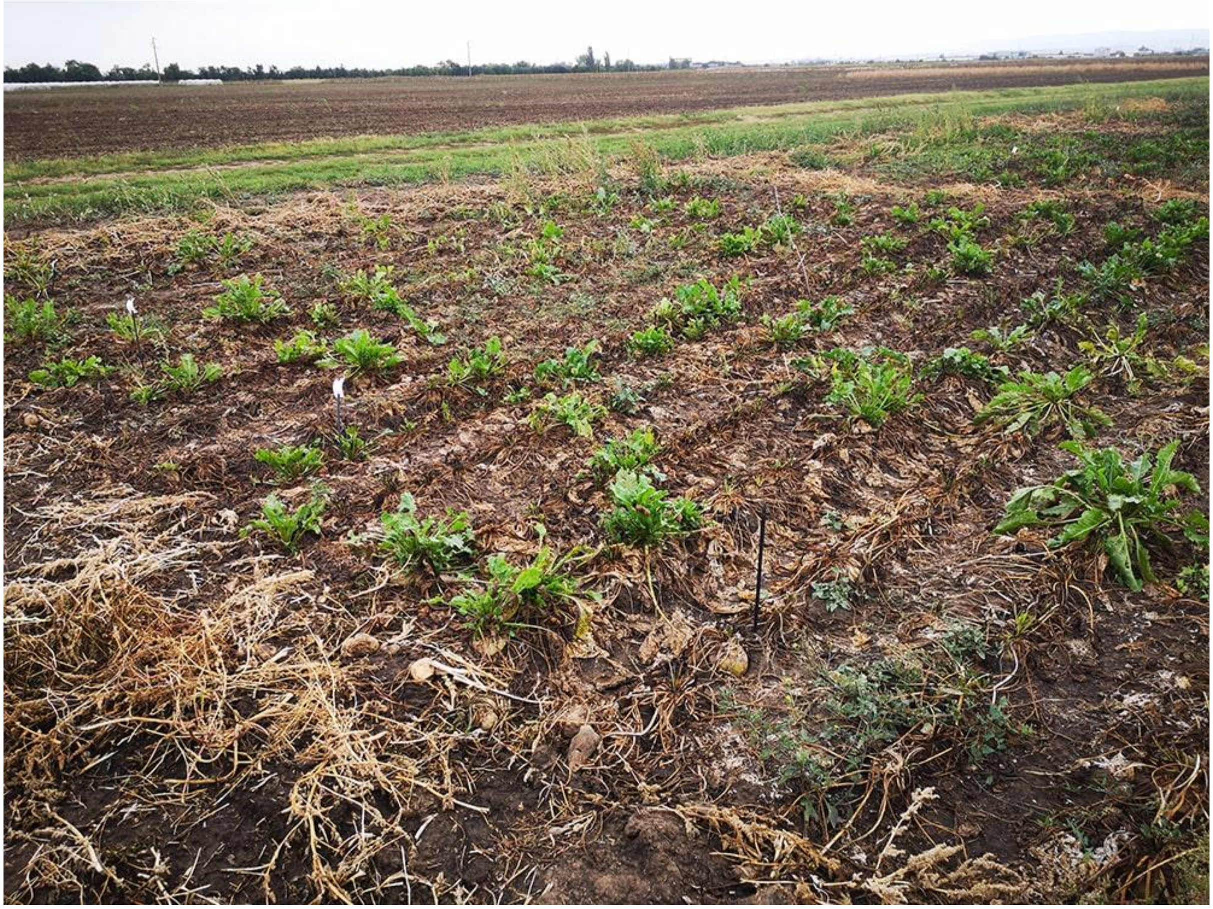

Supplementary Figure S2. Development of RTD symptoms in sugar beet experimentally inoculated with CaPsol by *R. quinquecostatus* (sensu Holzinger *et al.* 2003) in 2020*.*

(a) Experimentally inoculated sugar beet with progressive development of RTD leaf symptoms: yellowing, wilting and necrosis (left) and healthy sugar beet/negative control (right), photographed 30 DAI.

(b) The same experimental plants photographed 50 DAI. CaPsol-infected sugar beet (left) in a collapse phase.

(c) Variation in development of typical RTD leaf symptoms, yellowing and necrosis, expressing on sugar beets experimentally infected with CaPsol by *R. quinquecostatus.*

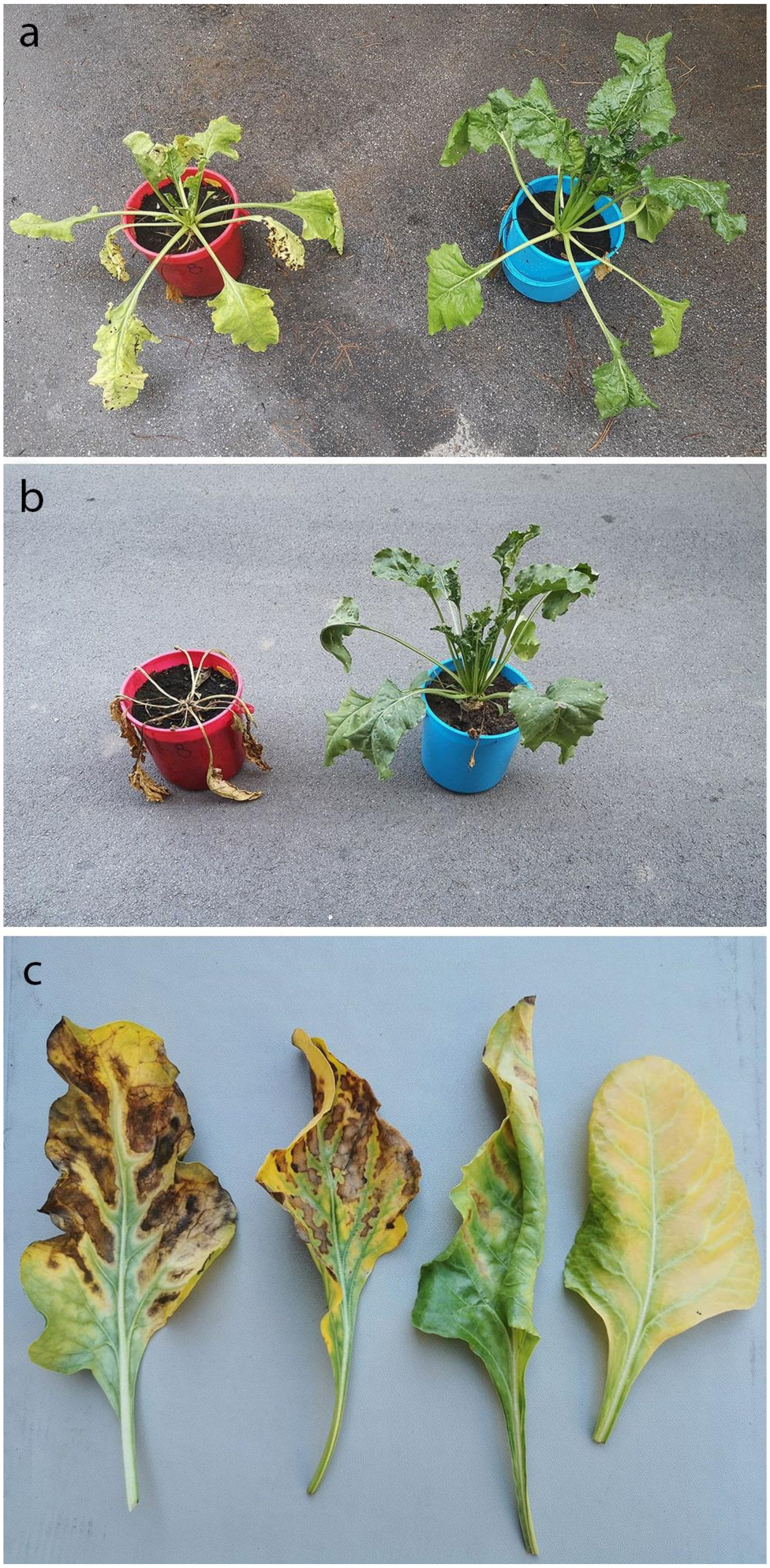

Supplementary Figure S3. Semi-field cage experiments set up on the experimental sugar beet plot-2 in Rimski Šančevi in 2021.

(a) Field net cages installed in plot-2 in May 2021, prior to the release of putative cixiid vectors.

(b) A field cage (coded "1") into which the population of *H. obsoletus ex C. arvensis* was released at the end of June, photographed in mid-August (45 DAI). The most of the sugar beet from the cage area, previously protected by the net, is in the collapse phase and was subsequently sampled.

(c) Negative control field cage (coded "2") photographed in mid-August (45 DAI) after removal of the cage net.

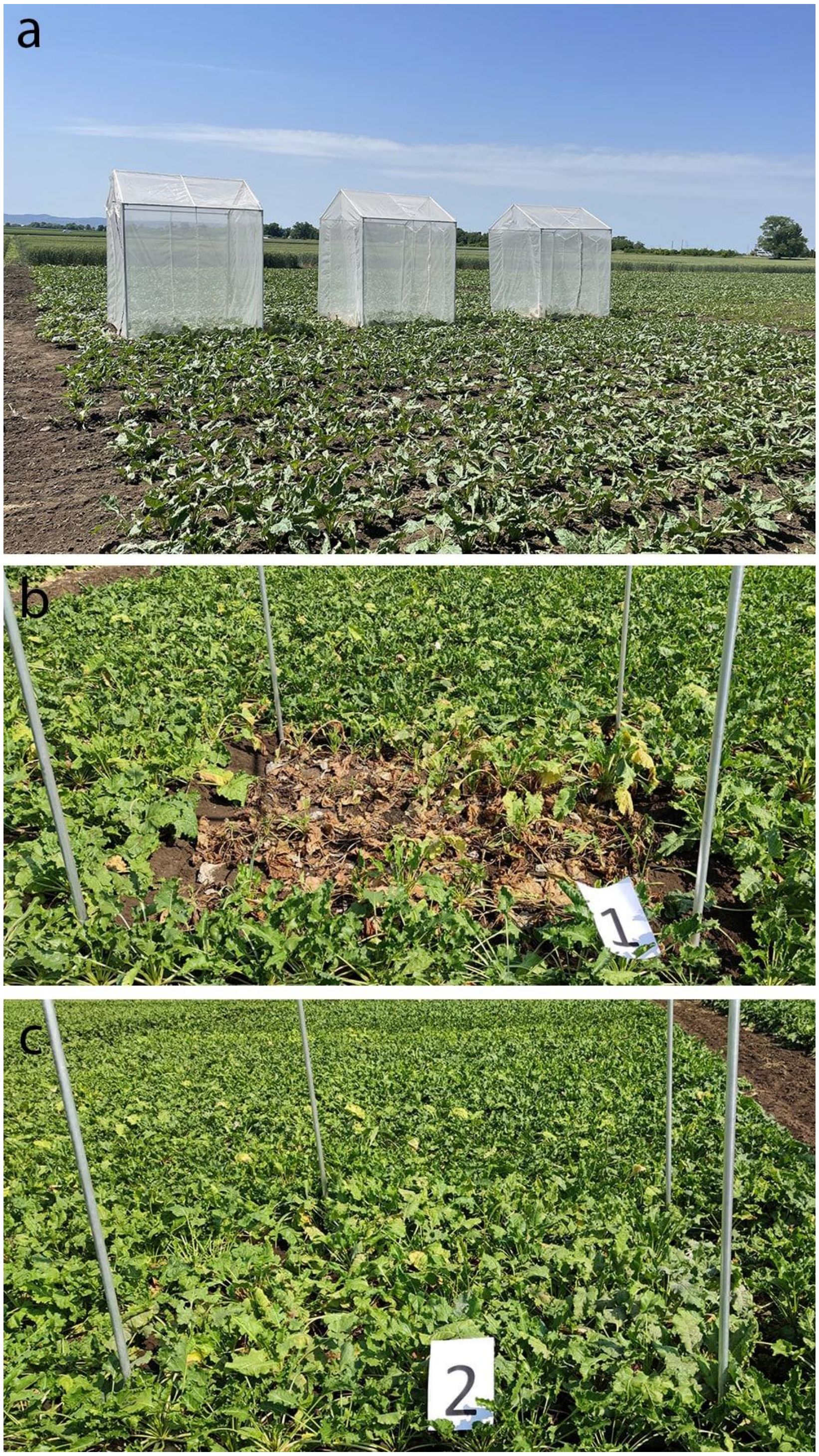

Supplement: Supplementary file 1 — Supplementary Information. [file 41598_2023_28562_MOESM1_ESM.pdf]
